# A Course of Clinical Lectures, Delivered at the Hôtel Dieu, Paris, for the Session 1842-’43

**Published:** 1843-09-02

**Authors:** A. F. Chomel

**Affiliations:** Paris


// THE MEDICAL EXAMINER,

And Retrospect of the Medical Sciences.

Vol. VI.] PHILADELPHIA, SATURDAY, SEPTEMBER 2, 1843. [No. 17.

### A COURSE OF CLINICAL LECTURES

*Delivered at the Hôtel Dieu, Paris, for the Session 1842–'43.*

BY A. F. CHOMEL, M. D.

### LECTURE XI.

RESUMÉ OF THE ANNUAL CLINIC.

(Continued.)

*Pneumonias and Pleurisies.*

In those cases which terminated favorably the mean duration was fourteen days; in the fatal cases the mean duration was twelve days. We noted the date of the disease at the period of entrance into the hospital. Those who recovered had been sick for five days; those that died seven days.

Among the most important symptoms or epiphenomena, we particularly noticed the influence of delirium upon the progress and termination of the disease. Delirium is sometimes connected with a phlegmasial affection of the brain, or its meninges, but very often it is symptomatic of some disease entirely distinct from the nervous centres or their membranes—as an affection of the stomach or lungs, for example. Three of our cases died with this symptom; the autopsies revealed no lesion of the brain, or its membranes. Wherever this symptom occurs in pneumonia the disease is grave; it announces a profound disturbance of the economy, against which nature is but too often unsuccessful.

Pneumonia is not unfrequently complicated with inflammation of the bronchial tubes, and with emphysema. This happens in four cases out of eleven or thirteen. We sometimes see jaundice occurring in the course of the disease; in itself it is an accident of but little moment, and is, in the majority of cases, purely symptomatic. In some cases the parotid glands tumify, suppurate, and in severe pneumonia, even pass into gangrene; I very lately saw a case in a young man, who recovered. In one case a very considerable pleuritic effusion occurred; this is a very serious complication, and the danger is imminent, as the patient might perish from asphyxia. In two instances we had articular rheumatism, complicating pneumonia. Both these cases recovered. You sometimes see pneumonia developed in women in a more or less advanced stage of pregnancy. In such cases there is a certain amount of risk, both for the mother and the child, for being obliged to treat the phlegmasia actively, abortion not unfrequently is produced. We had two cases of this kind; in both we were successful; that is to say that the mother was saved without the life of the child being endangered.

In twelve deaths out of eighty-one cases of pneumonia this year, we had four who were under fifty years of age. Now, this is a considerable mortality, for, as I remarked to you the other day, pneumonia is less fatal in middle age, *cæteris paribus.* It is important, therefore, to ascertain the causes which produced this mortality. Of these four fatal cases, one was a young man with a double pneumonia, accompanied with very serious symptoms; consequently it is not astonishing that he should have died. Another man, although in the flower of age, was an habitual drunkard; this was an aggravating circumstance, for there is nothing more certain than that parenchymatous phlegmasia in intemperate subjects, present great gravity. The third one was a woman of thirty-two years of age, convalescent from a severe dysentery, which she had had in the hospital. You can readily conceive that a pneumonia developed in an individual already enfeebled, by a previous malady, is under very unfavorable circumstances for resolution. It may be added, too, that this patient remained unattended for seven days, and that it was not until the eighth that any treatment was commenced. The fourth case was a man of forty-two years of age, who entered the hospital on the tenth day of the disease, and who previously had done nothing towards being treated. These four fatal cases, then, only confirm the principle that I have attempted to establish elsewhere—the slight mortality in pneumonia in youth and adult age.

*Treatment.*—The general considerations that I am now about to make, on the treatment of pneumonia, will complete the special remarks that I have had occasion to make on the various cases of pneumonia that have presented themselves to us during the present semester. Whenever an organ is inflamed, the first indication to fulfil is to place that organ in a state of absolute repose; this a therapeutic law which admits of no exceptions. In certain organs this is very easily accomplished; thus we can enforce entire rest in the case of a limb. But in other organs it is very difficult, and even, to a certain degree, impossible. Thus, how can we condemn the heart or the lungs to a condition of perfect rest—organs whose uninterrupted functions are necessary for the continuance of life? For these organs, therefore, complete repose is impossible. You can, however, diminish their activity up to a certain point, and that, 1st. By putting the body in a state of rest; by removing all exciting causes, whether moral or physical; in prescribing absolute silence. 2d. In diminishing, as much as possible, the mass of blood which flows towards these organs, and which gives them too much energy; and this is done by sanguine evacuations. Hence in pneumonia there is a direct indication to bleed; and it is principally by the subtraction of this exciting fluid, the blood, that we are enabled to procure for the lungs a state of comparative repose, and place them in a favourable condition for the resolution of the inflammation. To this heroic method we add, as adjuvants, diet, emollient drinks, etc. etc.

Bleeding is, therefore, the first and principal medication to employ against inflammation. On this point physicians generally agree; but what difference of opinion you will find when you come to establish some rule in the employment of this means. For a long time physicians have endeavoured to adopt some rule in this respect. Some preferred the morning for bleeding, others the evening; some every day, and others, again, several times in the day. Now, it is very difficult, not to say impossible, to establish any formula in such cases, because they necessarily must vary according to circumstances, as age, sex, constitution, and many other circumstances, which will vary to infinity. Besides, how many accidental circumstances may occur, which it is almost impracticable to foresee. So that we should reject all general rules respecting the number of times that we are to bleed. Some physicians limit themselves to one bleeding, afterwards resorting to other means. There are a few who proscribe it altogether. An example of this class may be found in Dr. Magendie, who supports his opinion not only by theoretical notions, derived from his experiments on animals, but also upon practical facts, observed in the service of his hospital. In spite, however, of the authority of this physician, I suspect that his observations are not yet sufficiently numerous to entitle his opinion to become a precept. For myself, I continue to bleed in pneumonia, relying on the accumulated experience of physicians in all ages.

The quantity of blood to be drawn varies very much. There are some individuals, who, independent of age, should be very sparingly bled; without this precaution they run the risk of suffering from dangerous prostration. Others, on the contrary, will bear large bleedings, and should have them. In certain individuals you must employ copious venesection, if you wish any amelioration; in others, again, the bleedings must be small, and repeated during the persistence of inflammation. I recollect a man of vigorous constitution, and sanguine temperament, whom we were able to bleed three times a day, and whose pneumonia lost none of its intensity until he had lost ten pounds of blood, in the space of four days. This man, whom every body thought would be exhausted by these copious venesections, was convalescent and walking about his room at the end of the tenth day. Such cases, it is true, are not common. In general, patients do not bear such numerous and large bleedings without becoming exceedingly prostrated. But, as I said before, you cannot establish any general rule in this respect; your practice must conform to the particular indications which may arise. It would certainly be a very happy circumstance to have, in place of these vague directions, some precise rule to follow; but such mathematical precision and exactitude are not to be found in our science. The same thing takes place in all human affairs, when one is obliged to conduct oneself after the laws of probability. Does not all human prudence consist in this? It is without doubt this which surrounds the exercise of our art with so many difficulties, and it is this which contributes also to exercise as well as to make manifest the genius of the practitioner.

In order to have a full appreciation of all the therapeutic indications, we should bear in mind a host of circumstances, some of the most important of which I am now going to touch upon.

1. *Stage of the Disease.* The precise stage of the disease is very important to know before we commence our treatment. Bleeding is particularly indicated in the first period of pneumonia, while the disease is on the increase. Many physicians think that this rule should be observed so strictly, that we should not bleed beyond the fourth or fifth day; this I think is carrying matters too far; there are many cases where, not only you may, but where you should bleed on the ninth or tenth day. I have already mentioned the resemblance pneumonia bears to erysipelas; like this affection it may successively be developed at various points of the pulmonary parenchyma, so that when one point is resolving, a neighbouring one may be about inflaming; so that in the same lung you may see at the same time various degrees of inflammation. Hence it results that where venesection is useless for one portion of the lung, where inflammation no longer exists, it may still be necessary for another part, where the disease is about commencing. For this as well as for the other reasons just mentioned, no precise rule can be given to regulate venesection; it would necessarily lead to error. In general we are more disposed to bleed at the commencement of the disease than at a later period, and properly so, but at the same time it is necessary to take into account the age and force of the patient, the antecedents of the disease, the state of the pulse, and a host of other circumstances which may offer a formal indication to bleed at an advanced stage of the disease,

2. *The extent of the Disease* is yet another circumstance to be considered, and which should guide us in the employment of sanguine evacuations. To determine with exactness the extent and intensity of the pneumonia, you should not restrict yourselves to the results obtained from auscultation, but you should refer to the state of the pulse as well as to all the other symptoms, both general and local, which could cast any light upon the diagnosis. How often has auscultation failed to reveal anything in a lung profoundly inflamed, and the reason is quite simple. By auscultation you may detect the state of the surface of the lungs, but never the condition of its deep seated portions; and every body knows that inflammation attacks often the centre of the organ, leaving untouched the superficies. In such cases the disease will be misunderstood, if not enlightened by other signs which the attentive physician should have before his eyes.

3. *Degree of Inflammation.* The physical signs are very precious in enabling us to appreciate this fact; for instance, when the lung offers to the ear of the observer bronchial respiration, with well marked crepitation, it is impossible to mistake the first stage of inflammation, the period at which venesection is the remedy *par excellence.* In such a case, should there be no counter indication, bleed more or less freely, and you will have good results. If there is only bronchial respiration without crepitant rale, let your venesection be less copious, for the disease has reached a more advanced period, and bleeding has less efficacy. Finally, if the respiration returns after several days without any rale, you may suspect suppuration, and you must then abstain from all sanguine evacuations, or use them with the greatest caution, if you would avoid plunging the patient in a state of extreme and dangerous prostration.

4. *The strength of the patient.* This is a point of the first importance to consider, in the employment of venesection. If your patient is vigorous, bleed freely, and you will probably not repent it. I mentioned to you a case where the blood-lettings were pushed to ten, in the space of four days with the greatest success; but it is important to know, and estimate correctly, the forces of patients, so as not to exceed the proper bounds, within which you may bleed with impunity. If, on the contrary, you have an individual feebly constituted, or weakened by disease or privation, or by misfortune and moral causes, you must be careful of his strength; a single bleeding in such a case is sufficient, and even then it should be a small one, made, as it were, exploringly. Pneumonia, like all the phlegmasiæ, has an ascensional progress, which should be appreciated; sometimes it appears to be arrested, and then suddenly it reappears with all the symptoms of the commencement. In such cases you should recur to venesection, unless there is some opposing indications. Because the disease has increased you should not imagine that the preceding bleedings have been useless; for venesection may modify the intensity of a phlegmasia, but it never succeeds in arresting its onward march.

6. *The age of the patient*, is a very important circumstance to be noted. Pinel, who practised in a large establishment for the old, has observed that venesection, in such cases of pneumonia, so far from being beneficial, frequently aggravated the disease. These results induced him to abandon sanguine evacuations. I was interne in his service for a year, and was enabled to verify the truth of his assertions. In spite of that, however, I believe that Pinel went too far, in laying it down as a law, that you should not bleed in pneumonia occurring in old persons. It is true that those who were taken ill at La Salpêtriére, where the air was foul, and where every species of hygienic care were wanting, and were in a very unfavourable condition, necessarily but illy supported blood-letting. But what an immense difference exists between them and those that now come under our care in the hospitals, where so many hygienic improvements have been introduced. What still greater difference between the old men of La Salpêtriére in the time of Pinel, and those old persons who reside in their own houses, surrounded by every moral and physical care. It is to be conceived that these last may be in a condition to support venesection. Besides, we frequently see old persons of good sound constitutions, who, when attacked by pneumonia, present a strong, full pulse, like men in the flower of life. In such cases why should we not bleed, if we do not exceed certain limits? After seventy years of age, for example, you should proceed with the greatest circumspection. One of the most important points in the treatment of pneumonia is to early distinguish real weakness from that which is only apparent. Apparent weakness proceeds from oppression of the forces by the disease, an oppression which ceases with the diminution of the quantity of the blood. The vessels are overloaded; by partially emptying them, you cause a return in the circulation to the normal state, and you give to the pulse a developement and fulness which before it did not possess. This oppression of the forces, this apparent feebleness, is rarely to be met with in old persons, but it occasionally occurs in those who possess a vigorous constitution. Thus, in some exceptional cases, even where there is apparent feebleness present, we may bleed old persons.

Such are the general rules which should guide us in the use of venesection in pneumonia. After bleeding, and as powerful adjuvants, should be placed laxatives. I employ them very often in these inflammations with success; they exercise a useful revulsion upon the intestinal tube, at the same time that they augment the secretion of the mucous membrane, which can but be advantageous, when the irritation is not too great.

Blistering is a therapeutic means of some value if employed with prudence, and at a proper time; but if prematurely used, the pulse becomes frequent, and the inflamed organ, instead of diminishing, is lighted anew. If, on the contrary, you apply blisters when the inflammation is on the decline, but when resolution is occurring slowly, you obtain good results. There is generally, to be sure, at first an increase of the general symptoms, a kind of fictitious fever; but at the end of thirty-four hours this declines, and a decided amelioration very often takes place. But, I repeat to you, that it is necessary to know how to seize the favourable moment for the application of this means. As to the size of the vesications, and the points to which they are to be applied, it is now generally received that they should be applied upon the chest, and preferably to that part which is over the seat of the inflammation, and that they should be large. When you apply blisters to the arm, and limit them to the size of a five franc piece, [a dollar] you cause as much suffering to your patients, and the results are much less sensible.

There is one mode of treatment which has been much extolled in pneumonia—large doses of tartar emetic. Some physicians give it from the commencement of the disease, without any preparative sanguine evacuation. It is in this manner that the school of Rasori employ it. For myself I use it differently. At first, whenever there is the indication for venesection, I bleed once or more, according to circumstances; then, when the acute stage has somewhat subsided, and resolution proceeds with difficulty, I frequently employ tartar emetic. But to do this the digestive canal should be in good condition, for if there be any intestinal irritation, or any particular susceptibility in that apparatus, that is a counter indication to the employment of the remedy given. With these conditions and with the precautions I have just mentioned, I have derived some advantage from it. What is its true action? For it has been variously interpreted by different schools. The Italians, partisans of Rasori, contend that tartar emetic acts in pneumonia as an antiphlogistic, by diminishing the energy of the vital forces, and consequently the inflammatory organism, and thus favours the resolution of the disease. I acknowledge that I do not comprehend this explanation, and I shall not stop to discuss the question, because I believe it to be a pure hypothesis. I prefer attributing to it a double action, more easily understood.

1. A revulsive action upon the digestive organs, by means of which the inflammation of the lung loses its intensity.

2. An action in some manner mechanical produced by vomiting, which brings into play all the muscles of the abdomen and chest, producing a kind of compression of the affected organ, which assists the resolution of the disease in the same manner that you dissipate, very often, erysipelas, by properly compressing the parts attacked. As to the real efficacy of the remedy, I believe it to be much less than its admirers would give to it. After the statistics of Dr. Grisolle, who has investigated this subject profoundly, it appears that of those who were treated by this means, one in three died, which is a very considerable mortality, supposing even that the cases in which this treatment was employed were really more severe than the others, it would still be a very great mortality. The facts then, neither give to this remedy an extraordinary efficacy, nor restrict its employment to the cases where there is a formal indication.

You sometimes have in pneumonia particular symptoms which impress at the same time a peculiar character and special indications. Thus we sometimes meet with that variety termed by Stoll bilious pneumonia. In this instance the emetic treatment, or rather the emeto-cathartic, is useful and even indicated. I have seen at the Hospital of la Charité, Bayle have great success with this treatment, and I have also employed it myself with great success. Again, you have pneumonia occurring with predominant adynamic symptoms; in such cases we are obliged to have recourse to the extract or wine of bark. These means you are obliged to employ from the commencement, because the disease, although inflammatory, is accompanied by such a state of debility, that antiphlogistic, debilitating treatment would be injurious. Tonics, too, are employed in those pneumonias which supervene at the termination of some other disease, or when the strength of the patient is already exhausted. In such cases, however, tonics have but little chance of doing good, for the organism is in such a state of debility that life seems all ready to leave it; whereas, in those cases where the pneumonia is primitive, and commences with the symptoms of adynamia, the use of tonics will frequently be followed by success.

Finally, this pulmonary inflammation may sometimes present itself with a train of ataxic symptoms very alarming. In such cases we must have recourse to musk, to castoreum, general baths, &c. Unhappily recovery in such cases is the exception.

In the course of an attack of pneumonia certain symptoms are developed, which will demand the especial attention of the physician, such as pain, cough, dyspnœa, etc.

1. *Pain.* This symptom requires serious consideration, when it is intense; the pain becomes, according to the axiom *ubi dolor, ibi fluxus*, the cause often having been the effect of the congestion and inflammation. You should seek early to cause it to disappear or to moderate its intensity. Several local bleedings, by leeches or cups, or a blister on the affected part, is ordinarily sufficient to remove it. The pain is sometimes so acute, that leeching or cupping have no effect, and we are then obliged to have recourse to large doses of opium. Sarcone employed this remedy in this manner, with great success in his practice, and he recommends it in his works. I have used it on the authority of this great physician with much benefit. But you should increase gradually the dose, and carry it as far as three or four grains, given in the course of the day in divided doses.

2. A *continued cough* is fatiguing. It is, however, rather an annoying than a dangerous symptom; and one which is best treated by mucilaginous and gummy potions.

3. *Dyspnœa* is a symptom which ordinarily occompanies pneumonia whilst it is on the increase, and it generally yields to the antiphlogistic measures directed against the inflammation itself. It offers, therefore, no particular indication to fulfil. Finally, there is one method which is rarely employed in hospitals, but which, nevertheless, is of great efficacy—I mean baths. The reason that they are so rarely employed in public establishments is that they are a therapeutic measure that requires great precaution, great care, and which would be far more injurious than useful, if the patients ran any risk of catching cold on leaving the bath. It is especially in private practice, where we can command every care, that this method is of such great advantage. It is especially useful in certain forms of pneumonia, when, for example, the skin is dry and burning, without the fever being very intense.

*Pleurisy.*—Pleurisy, when simple and uncomplicated with crepitation, is easy to diagnosticate, and it very often occurs under this form. Still it is not long since that physicians misunderstood it, and confounded it with inflammation of the substance of the lung itself. Whenever they had dulness, and some difficulty in the respiration, they immediately diagnosticated a pneumonia rather than a pleurisy. But such errors now a days are impossible, if one is at all skilled in physical exploration. Whenever you have dulness to any extent, you should suspect rather a pleurisy than a pneumonia, especially if there is none or very little fever, and if the dyspnœa is not considerable; for never, or hardly ever, do you see a pneumonia of any extent, without the pulse manifesting some febrile reaction, more or less pronounced, and that the respiration is more or less embarrassed. Besides, pleurisy is one of those affections which, differing from pneumonia, is disposed to become more or less chronic, either in its progress, or in the character and intensity of its symptoms. You frequently see a pleuritic effusion occurring suddenly, and without any prodrome; whilst pneumonia is almost always preceded by indications more or less characteristic, by more rapid progress, a more marked physiognomy, and by well-marked symptoms, as cough, dyspnœa, fever, &c. &c. Nothing then in reality is easier to distinguish, than a simple pleurisy from a pneumonia, whether the latter be frank or masked, or even chronic; the latter form, which is extremely rare, is the only one with which, to a certain extent, there is any chance of confusion.

Pleurisy itself is essentially an affection of little gravity. We have had this year nineteen cases, and all have recovered. I here count only those cases of simple pleuritic effusion, without any complication of the lungs or heart. Those cases of pleurisy consecutive to other disorders, which are on the contrary very dangerous, I shall consider hereafter in another group. We had the same number of pleurisies in the summer semester as in the winter, which constitutes another difference between this affection and pneumonia, which we have seen was more frequent in summer than in winter. No difference in regard to sex was observed this year. About thirty was the age at which the majority of cases happened. It is very difficult to determine the true cause of this disease. Cold, more especially sudden cold to the feet, whilst heated, is said to be the usual cause. We carefully questioned our cases on this point; twelve of them answered that they did not know to what to attribute their attack, and that they had not been suddenly chilled; four admitted this as a cause; and in one instance it followed small-pox. We have found it more frequently on the right than on the left side. Two-thirds were in the right pleura; one-third only in the left. In this fact there is an analogy between it and pneumonia. The extent of the disease was limited in sixteen cases. In these patients it occupied the inferior half or third of the chest; in some other instances two-thirds. In two cases the pleuritic effusion was so complete that the dulness extended from the clavicle. In spite of this the heart was not carried aside by the fluid; when there is deviation of the central organ of the circulation, the danger is great, and death very probable. Last year we had two cases of pleurisy with displacement of the heart, but both recovered, which is very rare.

Let us now examine consecutively the principal symptoms.

A *chill* at the commencement is very common; ten patients out of eighteen had this symptom.

*Pain in the side* is a very common phenomenon, being present in nearly all cases, with different degrees of intensity. This symptom is sometimes absent, however; the ancients then called it latent pleurisy. Out of eighteen cases it once manifested itself on the side opposite to the effusion. In all the cases but one the effusion existed at the period of the patient's entrance into the house; in that one it appeared subsequently. Auscultation and percussion always easily revealed to us the existence of effusion. In one-half the cases there was a feeble bronchial souffle; in two cases there was bronchial respiration well marked, as it appears in pneumonia; there might probably have been at the same time some parenchymatous inflammation of the lung. Vocal resonance, well marked egophony, occurred in eight cases; in six cases the voice was transmitted without being broken or its pitch raised; and in six cases there was no egophony. Ordinarily there is *egophonie de retour*, as the *rale crepitant de retour* in pneumonia. Sometimes this is wanting, especially when the pleuritic effusion lasts for some time. The cause of this is, that when the effusion has been protracted, the lung a long time compressed loses its elasticity, and returns with difficulty to its original condition; so that the phenomena of auscultation which depend on the return of the lung to its natural state, and the rapid diminution of the effusion, will necessarily be absent when these conditions are wanting. In three cases the pleuritic effusion was complicated with tubercles in the lungs; in spite of this complication it was resolved. Very often in the course of a tuberculous affection you see pleurisy developed, and rapidly disappearing and the organic affection continuing. Twice the pleurisy was complicated with slight pneumonia and intense bronchitis. In these instances the two diseases were resolved simultaneously. Twice it appeared in newly delivered women, and in them was complicated with abscess of the breast. This external affection did not influence the resolution of the pleurisy.

The treatment was the one most ordinarily employed. In severe cases we bled freely. In slight cases we used topical bleeding only. In addition we used purgatives, and demulcent drinks.

*Bronchitis.*—We had thirty-four cases of bronchitis, and three deaths. The relative frequency with regard to sex was very different from that which we have indicated in the preceding affections. In typhoid fever, pneumonia and pleurisy, we found men much more frequently attacked than women; now, in bronchitis the proportion is considerably greater. This year we had fourteen cases among men, and twenty among women.

Of all the forms, capillary bronchitis was the most grave. It was in this variety that the deaths took place. In one of the three fatal cases, a woman who, along with her bronchitis, presented a very rare and serious affection—a gangrenous vaginitis.

Bronchitis, in general, presents among other symptoms dyspnœa, a sibilant or sonorous rhonchus more or less marked, with sometimes considerable fever. In capillary bronchitis there is considerable dyspnœa, a fine crepitant rale, as in pneumonia, and very intense fever. The symptoms, which are those of a grave malady of the lungs, cannot be confounded with those of ordinary bronchitis; but, on the other hand, it is not so easy to distinguish it from other diseases of the lungs. In one of our cases the diagnosis was very obscure. This was a woman who presented an obscure sound at the summit of the lung, under the clavicles, with considerable vocal resonance, gargouillement. It was at first thought to be a tuberculous affection with excavation. There was violent fever. The symptoms rapidly increased, and the patient died. At the autopsy we found a number of small cavities, or kind of pouches, filled with purulent matter. At first it was thought to be softened tuberculous matter, but upon a more attentive examination it was found to be pus, or muco-purulent matter, contained in sacs formed by the dilated bronchi. The pulmonary parenchyma around these sacs was indurated, but no where was there any tuberculous matter. Here then was a case of chronic bronchitis, which was taken for a tuberculous affection, and which, in fact, presented symptoms which very easily might be misunderstood.

*Paris*, *May*, 1843.

